# Efficacy of the combined approach in treating major salivary gland stones

**DOI:** 10.1007/s00405-025-09704-2

**Published:** 2025-10-01

**Authors:** Jesper Stensig Aa, Joyce Dominique Horsmans Schultz, Lars Peter Schousboe

**Affiliations:** Department of Oto-Rhino-Laryngology Vejle, University Hospital of Southern Denmark, Beriderbakken 4, Vejle, 7100 Denmark

**Keywords:** Combined approach, Sialolithiasis, Salivary gland, Sialendoscopy

## Abstract

**Purpose:**

To evaluate the efficacy and safety of the combined approach in managing large salivary gland stones, with specific focus on stone elimination, symptom resolution, and postoperative complications.

**Methods:**

A retrospective cohort study was conducted at a tertiary referral centre, including patients diagnosed with sialolithiasis between 1 October 2018 and 1 October 2023. Patients treated with the combined approach were included; those managed solely with sialendoscopy, exclusively via incision, or conservative treatment were excluded. Medical records were reviewed for outcomes including stone removal success, symptom resolution, and complications.

**Results:**

Of 243 patients diagnosed with sialolithiasis, 33 underwent the combined approach - eight involving the parotid gland and 25 the submandibular gland. Complete stone removal was achieved in 28 cases (85%), and symptom resolution was observed in 29 cases (88%). Postoperative infections occurred in five parotid and four submandibular cases. Temporary tongue paraesthesia was noted in two submandibular cases; no nerve injuries were reported in parotid cases.

**Conclusion:**

The combined approach is a safe and effective gland-preserving technique for large salivary gland stones, with high success rates and minimal complications. Prophylactic antibiotics may be beneficial for parotid procedures, though prospective studies are needed to define infection criteria and guide antibiotic use.

## Introduction

Sialolithiasis is a common cause of inflammatory disease in the major salivary glands with an incidence of 5.5 cases per 100,000 persons per year [1]. When sialolithiasis results to ductal obstruction, patients typically experience with meal-related swelling and pain in the affected gland. Salivary stones vary in shape and size and may occur as single or multiple entities within a gland.

The advent of sialendoscopy in the late 1990 s for managing obstructive salivary gland diseases significantly transformed the treatment landscape for benign obstructive conditions in the major salivary glands [2]. Sialendoscopy enables direct visualization of the ductal system, facilitating the removal of salivary stones with specialized instruments or dilation of stenotic regions. Numerous studies have demonstrated sialendoscopy reduces the need for stone-related gland excision while supporting its safety and gland-preserving benefits [3, 4].

Current guidelines suggest that sialoliths up to five mm in diameter can be intraorally extracted using sialendoscopy, if freely situated within the ductal lumen [5]. For small, impacted stones, options include extracorporeal shock wave lithotripsy or laser fragmentation [6]. Conversely, larger stones present therapeutic challenges. Historically, partial or total parotidectomy or submandibulectomy were considered for severe symptoms. These interventions, however, come with risks, including potential nerve damage and Freys syndrome, and are often complicated by inflammation at the surgical site [3].

**Fig. 1:**
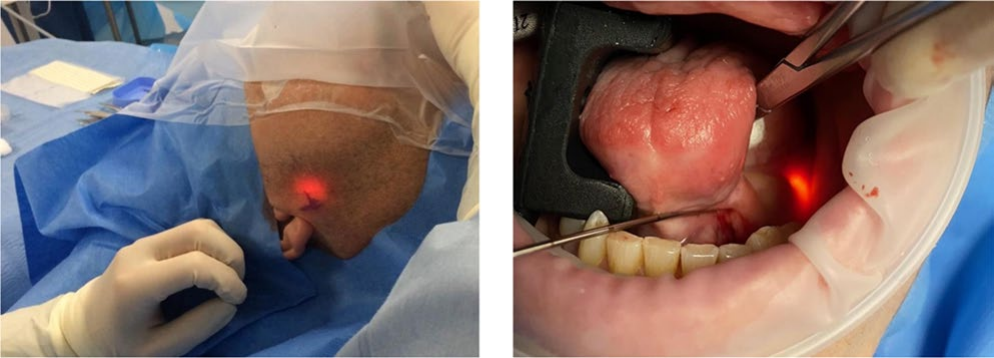
Showing transillumination from the sialendoscope, which guides the surgeon for stone extraction in both the parotid (left) and submandibular (right) gland

In contemporary practice, a minimally invasive combined approach has been used as a gland sparing alternative. This strategy combines endoscopic guidance to stabilize the stone with a targeted external intervention for extraction, using endoscopic transillumination for precise localization (Fig. 1). In the parotid gland, the approach involves a transcutaneous incision, while for the submandibular gland, it requires an intraoral incision. Previous research on the combined approach is limited by small sample sizes, highlighting the need for further studies [7–11].

The aim of this study was to evaluate the efficacy of the combined approach for treating large salivary gland stones, in terms of successful stone removal and the resolution of symptoms in patients. The occurrence of complications was also recorded.

## Materials and methods

This retrospective observational cohort study received exemption status from the Department of Regional Health Research and was approved by the institutional review board at the University Hospital of Southern Denmark (Acadre 23/1852).

Patients diagnosed with sialolithiasis at a tertiary referral centre over a five-year period were included in the initial cohort. From this group, patients treated with the combined approach procedure were identified, and their outcomes were analysed.

Medical records of patients diagnosed with sialolithiasis from October 1, 2018, to October 1, 2023, were reviewed. Inclusion was limited to patients who underwent the combined approach procedure for the major salivary glands. Exclusion criteria comprised patients treated solely by sialendoscopy, treated exclusively via incision, or managed conservatively. Referrals originated from private ENT specialists, other ENT departments or hospital departments within the region.

**Fig. 2:**
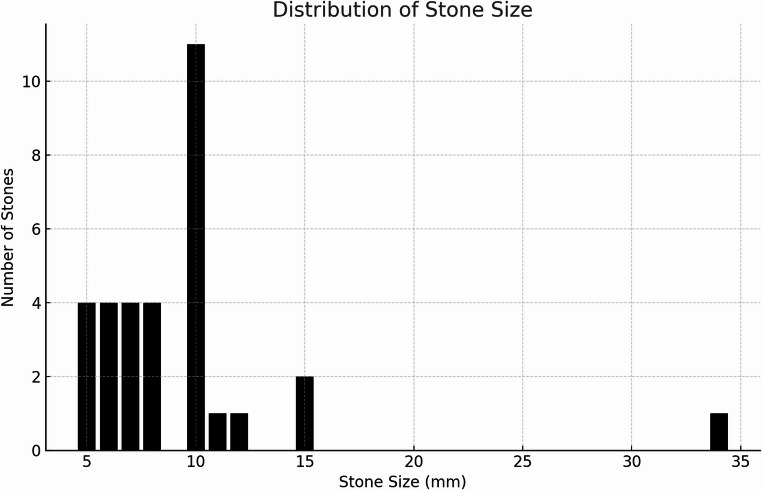
Distribution of stone size among patients undergoing the combined approach procedure

Medical records were systematically evaluated to assess stone elimination efficacy, symptom resolution, and the occurrence of any complications. Postoperative infection was defined as any instance in which antibiotic treatment was initiated following surgery, as documented in the medical records. Additional variables included patient demographics (gender, age), treatment indications, comorbidities, smoking status, medication use, imaging modality, stone size (Fig. 2) and location, follow-up duration, and any subsequent need for salivary gland excision.

Statistical analysis was performed using the statistical software R (version 4.5.0).

## Results

Among 243 patients diagnosed with sialolithiasis, 33 patients underwent the combined approach procedure: Eight cases involving the parotid gland and 25 involving the submandibular gland.

Stone elimination was achieved in 28 cases (85%): Seven cases (88%) in the parotid group and 21 cases (84%) in the submandibular group. One patient in the parotid group and three patients in the submandibular group presented with multiple stones.

Symptom resolution was observed in 29 cases (88%) following treatment: Six cases (75%) in the parotid group and 22 cases (88%) in the submandibular group. In the parotid group, two cases necessitated a subsequent partial parotidectomy, one of whom had an additional stone. For the submandibular group, two patients underwent gland excision during the same operation due to failure to remove the stone via the combined approach.

In the parotid group, five patients developed postoperative infections, with two requiring hospitalization. Postoperative infections were also observed in both cases that later required parotidectomy. All eight patients received a single 1500 mg dose intravenous cephalosporin (Cefuroxim B. Braun) perioperatively. The postoperative infection rate was significantly higher in the parotid group (5/8, 63%; 95% CI: 31–86%) compared to the submandibular group (4/25, 16%; 95% CI: 6–35%) (*p* = 0.02, Fisher’s exact test).

A logistic regression analysis was conducted to explore associations between patient characteristics and the occurrence of postoperative infection, using demographic and clinical variables as covariates. Only gender reached statistical significance (*p* = 0.05), with female gender being associated with an increased risk of postoperative infection.

No cases of nerve damage, including facial nerve injury, were observed.

In the submandibular group, postoperative infections occurred in four patients, with one requiring hospitalization. One of these patients, who underwent gland removal, was managed as an outpatient. Two patients experienced temporary tongue paraesthesia. One patient developed ductal stenosis but declined further intervention. Additionally, three patients later presented with a gland that felt firmer on palpation.

Ultrasound was performed in all 33 patients preoperatively. One patient underwent an additional CT scan due to inconclusive ultrasound findings.

## Discussion

In this study, 33 patients with sialolithiasis were treated using the combined approach procedure. Outcomes were evaluated in terms of technical success, defined as complete stone removal, and clinical success, defined as symptom resolution. These endpoints were reported separately to provide a comprehensive assessment of procedural efficacy and patient-centered outcomes.

Stone removal was achieved in 28 cases overall (88% in the parotid group and 84% in the submandibular group), while symptom resolution was observed in 29 cases (75% and 88%, respectively). However, these two measures did not always align. In the parotid group, one patient continued to experience symptoms despite initial stone removal and subsequently underwent a partial parotidectomy. Intraoperative findings revealed the presence of an additional, previously undetected stone within the gland. In the submandibular group, two patients underwent gland excision during the same surgical procedure after unsuccessful stone retrieval due to distal fixation. Both became symptom-free, yet these cases were not counted as technical successes. In another case, the stone could not be removed, and gland excision was not pre-consented; however, the patient later reported spontaneous stone expulsion with symptom relief, possibly due to ductal dilation during sialendoscopy. Another patient developed ductal stenosis but declined further intervention due to mild symptoms.

These cases underscore that while complete stone removal remains the primary goal of the combined approach, it does not always correlate with symptom resolution. Therefore, both endpoints are clinically relevant and should be considered in future prospective studies, ideally using standardized criteria for procedural success that capture both surgical and symptomatic outcomes.

Jadu et al. [12] conducted a systematic review of the combined approach for both the parotid and submandibular glands. Including a total of 409 patients, they found the combined approach to be safe, with successful stone removal rates ranging from 69 to 100% and a pooled success rate of 92.8%.

Roland et al. [9] performed a systematic review and meta-analysis of patients who underwent the combined approach for the parotid gland. The analysis included 184 patients, with a weighted success rate of 99% for stone removal and 97% for symptom improvement.

These findings are consistent with the results of our study.

In this study, no major complication was observed with the combined approach. Notably, no damage to the facial or lingual nerve was observed.

Postoperative infections occurred in five of eight patients in the parotid group (63%). All patients in this group received a single dose of 1500 mg intravenous cefuroxime as perioperative prophylaxis. Despite this, two patients required hospitalization for intravenous antibiotic treatment, and three others were managed with oral antibiotics. This infection rate is considerably higher than those reported in previous studies. Given the retrospective nature of the study, it is difficult to confirm the accuracy of the infection diagnoses. Postoperative discomfort such as pain and salivary swelling is expected following the combined approach and may have been mistaken for infection in some cases.

In comparison, four patients in the submandibular group developed postoperative infections, with one requiring hospital admission. None of the patients in this group received prophylactic antibiotics perioperatively. This difference in antibiotic use reflects local clinical practice rather than adherence to a standardized protocol.

At present, there are no widely accepted guidelines that specifically address the use of antibiotics in relation to the combined approach procedure. In Denmark, the use of antibiotics is deliberately conservative, guided by national strategies aimed at reducing antimicrobial resistance. The findings of this study highlight the need for future prospective research to clarify the role of perioperative and postoperative antibiotic treatment. Such studies should aim to define infection criteria more clearly and to develop standardized treatment protocols, especially for procedures involving the parotid gland where the risk of infection may be higher.

A logistic regression analysis was conducted in this study to evaluate whether specific patient or stone characteristics could predict the risk of postoperative infection. While gender emerged as a statistically significant factor, with females showing a higher likelihood of infection, the clinical relevance of this finding is limited due to the small sample size and a low explanatory power (low R² value) of the model. As such, this finding should be interpreted with caution and warrants further investigation in larger, prospective cohorts.

Other complications were minor. Two patients experienced temporary lingual nerve symptoms. The lingual nerve is in close relation to the Whartins duct and is therefore exposed when a big stone must be removed. Additionally, three patients had a palpable firmer submandibular gland, possibly due to postinfectious fibrosis.

In this investigation, a cohort of 25 patients underwent a combined approach procedure on the submandibular gland. Not all centers have access to sialoendoscopes, which is an important consideration. A comparative study by Coca et al. [13] assessed the efficacy of the combined approach versus incision alone. This study concluded that the use of sialoendoscopy does not significantly enhance patient outcomes. Given the high cost and fragility of sialoendoscopes, which are susceptible to damage even during routine cleaning, their use may not be cost-effective in all cases. Therefore, it may be prudent to reserve sialoendoscopy for specific submandibular gland cases where the stones are impalpable or mobile. A notable advantage of sialoendoscopy is its ability to inspect the duct post-stone removal, offering the opportunity to detect multiple stones if present.

In experienced hands, ultrasound is an excellent radiological tool for detecting sialolithiasis. Only one patient required a supplemental CT scan due to the clinical suspicion of a stone not identified on ultrasound, aligning with findings from other studies [14]. In this study, all patients had a stone size bigger than five mm, and thus visible on ultrasound. In more complex cases, cone beam computed tomography (CBCT), or magnetic resonance imaging (MRI) may be considered as adjunctive imaging modalities. CBCT offers high-resolution, three-dimensional visualisation with relatively low radiation exposure and is particularly beneficial for identifying non-calcified or deeply situated calculi. MRI, while less commonly employed, may aid in assessing ductal pathology or distinguishing sialolithiasis from other soft tissue conditions when inflammation or neoplasia is suspected.

A strength of this study is its inclusion of 243 patients diagnosed with sialolithiasis, providing a robust dataset for examining the effectiveness of the combined approach procedure. Among these, 33 patients underwent the combined approach, a selection based on the criterion that only patients with stones larger than five mm were treated (Fig. 2). The demographic and clinical characteristics of the study participants, covering a wide age range (15–72 years) and various medical conditions, enhance the generalizability of the findings (Table 1).

**Table 1:** Demographic and clinical characteristics of study participants (*n* = 33) medical conditions

Characteristic	Value
Age (years), median (range)	50 (15–72)
Gender (male: female)	15:18
Smoking status	Active: 12 Former: 3 Never: 18
Follow-up period (months), median (range)	4 (1–18)
Stone size (mm), median (range)	10 (5–34)
Stone location (cm from papilla), median (range)	5 (2–7)
Condition	Number of Participants
Charlson Comorbidity Index, median (range)	1 (0–4)
Cardiovascular disease	4
Autoimmune disease	4
Pulmonary disease	5
Endocrine disease	9
Antipsychotic drugs	2
Antiepileptic drugs	1
Antihypertensive drugs	10

A limitation of this study is its retrospective nature, relying on outcomes recorded in medical records. Given the relatively short median follow-up duration of 4 months (range: 1–18), the study may underestimate the incidence of late recurrences or delayed complications, which could impact long-term outcome assessments.

## Conclusion

The combined approach is an effective gland-sparing technique for the management of major salivary gland stones. The procedure demonstrates high success rates with relatively few complications, making it a valuable addition to the therapeutic options for sialolithiasis. It offers a favourable balance between efficacy and patient safety.

Based on our findings, prophylactic postoperative antibiotics may be advisable for patients undergoing the combined approach in the parotid gland. However, further prospective studies with clearly defined criteria for diagnosing postoperative infections are needed to confirm this recommendation.
